# Pain in HIV Patients

**DOI:** 10.4103/0973-1075.63137

**Published:** 2010

**Authors:** Wiwanitkit Viroj

**Affiliations:** Wiwanitkit House, Bangkhae, Bangkok, 10160 Thailand

Sir,

I read the recent publication by Nair *et al*,[1] with great interest. I agree with the authors that the problem of pain that needs palliative care among HIV/AIDS patients exists. However, the author has focused on a rather severe group of HIV infected patients and this leads to the finding that pain is common. My query is whether there is any selection bias on this work and if the conclusion can be applied for all HIV infected subjects.
